# Hippocampal PPARα Plays a Role in the Pharmacological Mechanism of Vortioxetine, a Multimodal-Acting Antidepressant

**DOI:** 10.3389/fphar.2021.673221

**Published:** 2021-06-15

**Authors:** Yuan Wang, Jiang-Hong Gu, Ling Liu, Yue Liu, Wen-Qian Tang, Chun-Hui Ji, Wei Guan, Xin-Yi Zhao, Ying-Fang Sun, Da-Wei Xu, Bo Jiang

**Affiliations:** ^1^ Department of Pharmacology, School of Pharmacy, Nantong University, Nantong, China; ^2^ Provincial Key Laboratory of Inflammation and Molecular Drug Target, Nantong, China; ^3^ Department of Orthopaedics, Affiliated Hospital 2 of Nantong University, Nantong, China

**Keywords:** chronic unpredictable mild stress, chronic social defeat stress, depression, peroxisome proliferator activated receptor α, vortioxetine

## Abstract

As a well-known multimodal-acting antidepressant, vortioxetine is thought to aim at several serotonin (5-HT) receptors and the 5-HT transporter. However, recently more and more proteins besides 5-HT are being reported to participate in the antidepressant mechanism of vortioxetine. As a widely known nuclear hormone receptor, peroxisome proliferator activated receptor *α* (PPARα) possesses transcriptional activity and is very important in the brain. Several reports have suggested that hippocampal PPARα is implicated in antidepressant responses. Here we speculate that hippocampal PPARα may participate in the antidepressant mechanism of vortioxetine. In this study, chronic unpredictable mild stress (CUMS), chronic social defeat stress (CSDS), behavioral tests, the western blotting and adenovirus associated virus (AAV)-mediated gene knockdown methods were used together. It was found that vortioxetine administration significantly reversed the inhibitory actions of both CUMS and CSDS on the hippocampal PPARα expression. Pharmacological blockade of PPARα notably prevented the antidepressant actions of vortioxetine in the CUMS and CSDS models. Moreover, genetic knockdown of PPARα in the hippocampus also significantly blocked the protecting effects of vortioxetine against both CUMS and CSDS. Therefore, the antidepressant effects of vortioxetine in mice require hippocampal PPARα.

## Highlights

1) Vortioxetine treatment ameliorated the down-regulating effects of both CUMS and CSDS on hippocampal PPARα.
2) Pharmacological blockade of PPARα abolished the protecting effects of vortioxetine against both CUMS and CSDS.
3) Genetic knockdown of hippocampal PPARα also prevented the protecting actions of vortioxetine against both CUMS and CSDS.

## Introduction

Depression is a chronic, recurring, and debilitating mental illness. People who have major depressive disorder (MDD) experience various psychological symptoms including feeling of hopelessness, sadness, lack of motivation, and difficulties to concentrate (Gartlehner et al., 2017; Sheehan et al., 2017). Due to multifactorial nature and heterogeneous symptomatology, the precise etiology of MDD remains elusive (Sheline et al., 2019). It is known that chronic stress and psychosocial trauma are prevalent determinants of MDD. In particular, adverse events in early-life increase the vulnerability to chronic stress and facilitate the development of MDD later in life (Vergne and Nemeroff, 2006; VanTieghem and Tottenham, 2018). Current antidepressants used in clinical are mostly designed to modulate monoaminergic neurotransmitters (Pereira and Hiroaki-Sato, 2018). These drugs are widely used to treat MDD and relatively safe. However, an elusive phenomenon is that the therapeutic actions of monoaminergic antidepressants always require weeks or even months of administration to produce (Alamo and López-Muñoz, 2009; Blier and El Mansari, 2013). The discrepancy between the acute neurochemical effects and clinical efficacy of monoaminergic antidepressants has puzzled many researchers and reminded us that depression neurobiology is complex and far from elucidated, needing further research (Vidal et al., 2011; Pilar-Cuéllar et al., 2013).

Peroxisome proliferator activated receptor α (PPARα) is widely distributed in both the peripheral and central nervous system tissues (Grygiel-Górniak, 2014). Early investigation and characterization of PPARα suggested a primary physiological role of this protein in lipid and lipoprotein metabolism and the regulation of inflammatory processes (Gervois and Mansouri, 2012; Bougarne et al., 2018). Recently, it has been demonstrated that PPARα is also involved in the pathogeneses of many neurological disorders such as stroke, Alzheimer’s disease, Parkinson’s disease and epilepsy (Bordet et al., 2006; Fidaleo et al., 2014; D'Orio et al., 2018). We previously reported that chronic stress down-regulated both the protein and mRNA expression of PPARα in the hippocampus, while did not affect PPARα in other brain regions such as medial prefrontal cortex (mPFC), amygdale, nucleus accumbens (NAc), ventral tegmental area (VTA) and hypothalamus (Song et al., 2018). Both genetic overexpression and pharmacological activation of PPARα in the hippocampus fully protected against chronic stress, producing significant antidepressant-like actions in mice (Jiang et al., 2015; Jiang et al., 2017; Ni et al., 2018; Song et al., 2018). In contrast, genetic knockout and knockdown of PPARα aggravated depression in mice (Song et al., 2018). Moreover, hippocampal PPARα was involved in the antidepressant mechanism of fluoxetine (a well-known selective serotonin (5-HT) reuptake inhibitor (SSRI)) (Song et al., 2018). Therefore, hippocampal PPARα is implicated in both the pathogenesis of depression and antidepressant responses.

As a multimodal-acting antidepressant, vortioxetine is thought to antagonize 5-HT_3_, 5-HT_7_ and 5-HT_1D_ receptors, activate 5-HT_1B_ and 5-HT_1A_ receptors, and inhibit 5-HT transporter (SERT) (Pehrson et al., 2016). Vortioxetine was approved by Food and Drugs Administration (FDA) for the treatment of MDD in 2013. Vortioxetine has also been reported to have promoting effects on learning and memory (Bétry et al., 2015; Frampton, 2016). Although thought to aim at the 5-HT system, recently more and more proteins besides 5-HT are being found to participate in the antidepressant mechanism of vortioxetine. Here we have a hypothesis that hippocampal PPARα may be involved in the antidepressant actions of vortioxetine. In this study, various methods and tests were used together to explore this speculation.

## Materials and Methods

### Animals and Ethical Statements

Adult male C57BL/6J mice (8 weeks old) were bought from SLAC Laboratory Animal Co., Ltd. (Shanghai, China). Adult male and female CD1 mice (50 weeks old) were got from the experimental animal center of Nantong University. Before use, the experimental mice were housed in groups (5 per cage) for 1 week to allow acclimatization. The experimental mice were kept at 23–25°C under a 12:12 h light/dark cycle with free access to food and water (55 ± 10% relative humidity; noise less than 50 db; ammonia concentration less than 14 mg/m^3^; bedding replacement twice a week). The behavioral experiments were carried out during the light phase. For animal sacrifice, all mice were anaesthetized using carbon dioxide and then killed by cervical dislocation. The experiment procedures involving animals and their care were conducted in accordance with the ARRIVE guidelines (Kilkenny et al., 2010; McGrath and Lilley, 2015), and approved by the Animal Welfare Committee of Nantong University (Approval No. 20180149–001).

### Materials

Vortioxetine and MK886 were provided by Targetmol (Boston, MA, United States). GW6471 was provided by Tocris (Bristol, United Kingdom). The vehicle for vortioxetine, GW6471 and MK886 was 1% DMSO in 0.9% saline. The dosages of vortioxetine (10 mg/kg), GW6471 (1 mg/kg) and MK886 (3 mg/kg) in this study were determined according to published reports (Zuena et al., 2018; Witt et al., 2019). All drugs were intraperitoneally (i.p.) injected in a volume of 10 ml/kg.

### Chronic Unpredictable Mild Stress

As we previously described(Ni et al., 2018; Xu et al., 2018; Jiang et al., 2019; Wang et al., 2020), in brief, 8 stressors were adopted in this study: damp bedding (24 h), cage tilting (12 h), restraint (1 h), shaking (30 min), 4°C exposure (1 h), day/night inversion, food deprivation (23 h) or water deprivation (23 h). All these stressors were randomly given for 8 weeks, and administration of vortioxetine/GW6471/MK886/vehicle was performed daily during the last 2 weeks. Afterwards, the FST, TST and sucrose preference test were performed together to assay the depressive-like behaviors of animals. The non-stressed control mice were left undisturbed except general handing (e.g. regular cage cleaning) and drug treatment.

### Chronic Social Defeat Stress

As we previously described (Jiang et al., 2015; Jiang et al., 2017; Xu et al., 2018; Jiang et al., 2019; Wang et al., 2020), briefly, each C57BL/6J mouse was exposed to a different male CD1 aggressor mouse for up to 10 min each day for a total of 10 days. When the social defeat session ended, the resident CD1 mouse and the intruder C57BL/6J mouse were each housed in one half of the cage and separated by a perforated Plexiglas divider for the remainder of a 24-h period. To minimize harm, when the C57BL/6J mice displayed submissive behaviors including immobility, crouching, trembling, fleeing and upright posture, the dividers were immediately set. On day 11, all defeated C57BL/6J mice were housed individually and injected daily with vortioxetine/GW6471/MK886/vehicle for another 2 weeks. Afterwards, the FST, TST, SPT and social interaction test were performed to detect the depressive-like behavior of animals. The non-stressed control mice were left undisturbed except general handing (e.g. regular cage cleaning) and drug treatment.

### Forced Swim Test

In brief, the mice were individually placed in a transparent glass tank (containing 15 cm high pure water, 25 ± 1 °C) for 6 min. A stopwatch was used to record the duration of immobility for each mouse during the last 4 min. The water was replaced after each trial. The immobility of each mouse was defined as it was floating in the water without struggling or having only slight movements to keep its nose above the water. This test was recorded with the observer unaware of the experimental grouping.

### Tail Suspension Test

In brief, the tail tip of each mouse was individually glued to a rail 60 cm above the floor, and hung for 6 min. The immobility (completely motionless) duration of each mouse during the 6-min period was recorded. This test was recorded with the observer unaware of the experimental grouping.

### Sucrose Preference Test

The test C57BL/6J mice were individually housed and allowed to drink 1% sucrose solution and pure water freely. Before the test, a process of sucrose preference training was performed. Each test mouse was given one bottle of pure water and one bottle of 1% sucrose solution for 2 days, during which the positions of two bottles was exchanged every 6 h to avoid side preference. On third day, the test mice were deprived of food and water for 18 h. On fourth day, each mouse was given two pre-weighed bottles for 6 h. After that, two bottles were weighed again. The sucrose preference index was calculated as a percentage of the consumed sucrose solution relative to the total amount of liquid intake.

### Social Interaction Test

This test comprises two trials for 5 min each. Briefly, an experimental C57BL/6J mouse was placed into a white plastic box (50 × 50 × 45 cm) containing an empty wire mesh cage (9 × 9 × 10 cm) positioned against the wall and allowed to explore for 5 min (target absent). Then, the C57BL/6J mouse was returned to its home cage for 1 min, while an unfamiliar male CD1 mouse was placed inside the wire mesh cage. Next, the C57BL/6J mouse was placed into the box again and allowed to explore for another 5 min (target present). The duration of time spent in the interaction zone (5 cm wide area around the wire mesh cage) for each trial was recorded by an investigator blind to the groups. The equipment was cleaned with 70% ethanol and dried between trials.

### Western Blotting

This method was performed according to previous studies with slight modifications (Zhang et al., 2011; Liu et al., 2012; Li et al., 2013; Wang et al., 2015; Xue et al., 2019). After sacrifice, the whole brains were removed. The hippocampus tissues of each mouse were individually dissected and homogenized in NP-40 lyses buffer containing PMSF (100:1). After centrifugation (12,000 rpm × 15 min, 4°C), the supernatants were collected and denatured. The BCA method was adopted to determine protein concentrations. The western blotting procedures were performed in a common way: 1, SDS-PAGE separation; 2, proteins transfer; 3, proteins blocking; 4, TBST washing; 5, Primary antibodies incubation; 6, TBST washing; 7, Secondary antibodies incubating; 8, TBST washing; 9, membranes scanning. Primary antibodies against PPARα (1:500; Abcam, Bristol, United Kingdom) and β-actin (1:500; Cell Signaling, Danvers, MA, United States) were used. IR-Dye 680-labeled secondary antibodies (1:5000; Licor, Lincoln, United States) were also used. An Odyssey CLx detection system was adopted for scanning.

### Adenovirus Associated Virus-Mediated Gene Knockdown

The production of AAV-PPARα-short hairpin RNA-enhanced green fluorescence protein (AAV-PPARα-shRNA-EGFP; Genechem, China) has been described in our previous study (Song et al., 2018). In brief, each mouse was individually anesthetized with 0.5% pentobarbital sodium and fixed in a stereotactic frame. After making a small drill hole on the skull of each mouse, a 10 µL Hamilton syringe was positioned at the following coordinates: AP = - 2.3 mm, ML = ± 1.6 mm, DV = + 1.8 mm. AAV-PPARα-shRNA or AAV-Scrambled-shRNA was bilaterally infused into the hippocampus region of each mouse using the syringe at a rate of 0.5 µl/min (1.5 µl/each side). After the infusion, the syringe was left in place for 5 min before being retracted slowly. The wound of each mouse was cleaned and sutured. 2 weeks were required for the expression of AAV to be stable in the hippocampus. The titers of AAV-PPARα-shRNA and AAV-Scrambled-shRNA were adjusted to 5 × 10^12^ TU/ml before use. The sequences for PPARα-shRNA and Scrambled-shRNA were 5′-AGA​AAT​TCT​TAC​CTG​TGA​A-3′ and 5′-TTC​TCC​GAA​CGT​GTC​ACG​T-3′ respectively (Song et al., 2018).

### Statistical Analysis

The SPSS Statistics 26.0 software was adopted for statistical analyses. Multiple group comparisons were performed using two-way ANONA followed by Bonferroni’s test. All data are expressed as means ± S.E.M, and a value of *p* < 0.05 was considered statistically significant.

## Results

### Repeated Vortioxetine Treatment Fully Reversed the Decrease in Hippocampal PPARα Expression Induced by Both Chronic Unpredictable Mild Stress and Chronic Social Defeat Stress

**FIGURE 1:**
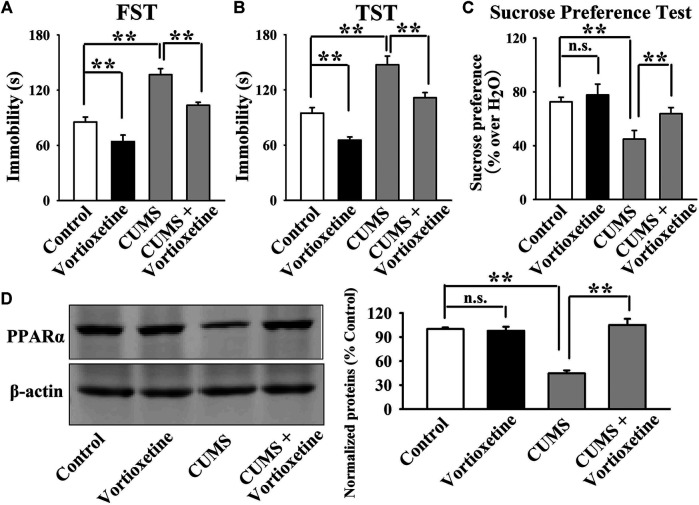
The CUMS-induced decrease in the hippocampal PPARα expression was significantly reversed by repeated vortioxetine treatment. **(A–C)** The antidepressant effects of vortioxetine in the CUMS model, as detected by the FST, TST and sucrose preference test. C57BL/6J mice subjected to 8 weeks of CUMS were daily injected with vehicle/vortioxetine during the last 2 weeks, and then subjected to behavioral tests. **(D)** Representative images and quantitative analyses indicate that vortioxetine administration fully antagonized the inhibitory effects of CUMS on the hippocampal PPARα expression. For **(A–C)**, *n* = 10 per group; For **(D)**, *n* = 5 per group. The data are expressed as the means ± S.E.M.; **p < 0.01; n.s., no significance. The comparisons were made by two-way ANOVA followed by Bonferroni’s test.

**FIGURE 2:**
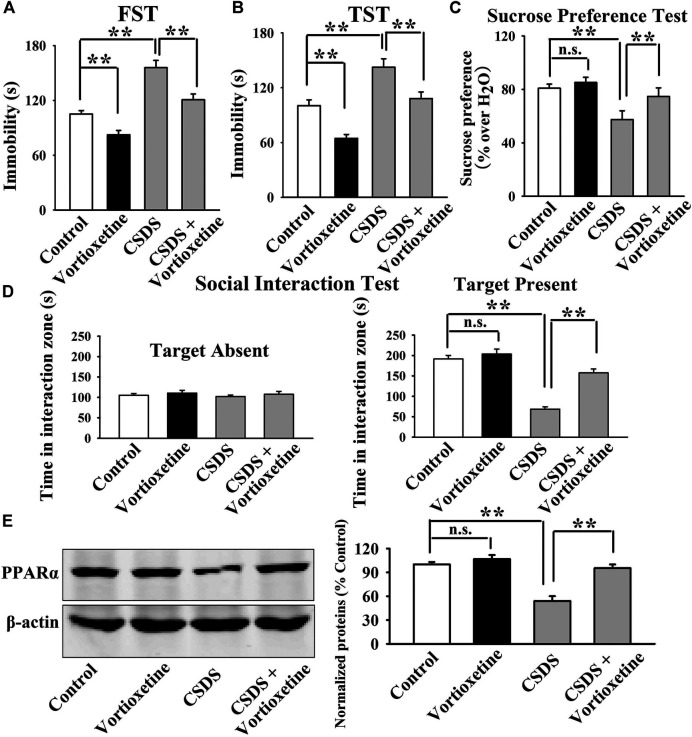
The CSDS-induced decrease in the hippocampal PPARα expression was fully restored by repeated vortioxetine treatment. **(A–D)** The antidepressant effects of vortioxetine in the CSDS model, as detected by the FST, TST, sucrose preference test and social interaction test. C57BL/6J mice subjected to 10 days of CSDS were daily injected with vehicle/vortioxetine for another 2 weeks, and then subjected to behavioral tests. **(E)** Representative images and quantitative analyses show that vortioxetine administration notably prevented the inhibitory effects of CSDS on the hippocampal PPARα expression. For **(A–D)**, *n* = 10 per group; For **(E)**, *n* = 5 per group. The data are expressed as the means ± S.E.M.; **p < 0.01; n.s., no significance. The comparisons were made by two-way ANOVA followed by Bonferroni’s test.

The antidepressant actions of vortioxetine were first examined in the CUMS and CSDS models. As shown in Figures1 A–C, repeated vortioxetine injection significantly ameliorated not only the enhancing effects of CUMS on mice immobility in the FST [ANOVA: CUMS, F(1, 36) = 58.935, *p* < 0.01; Vortioxetine, F(1, 36) = 20.874, *p* < 0.01; Interaction, F(1, 36) = 1.073, *p* = 0.307] and TST [ANOVA: CUMS, F(1, 36) = 58.050, *p* < 0.01; Vortioxetine, F(1, 36) = 25.487, *p* < 0.01; Interaction, F(1, 36) = 0.216, *p* = 0.645] but also the down-regulating effects of CUMS on the sucrose preference [ANOVA: CUMS, F(1, 36) = 12.758, *p* < 0.01; Vortioxetine, F(1, 36) = 5.714, *p* < 0.05; Interaction, F(1, 36) = 1.377, *p* = 0.248] of mice (*n* = 10). Similarly, Figures 2A–D indicate that repeated vortioxetine injection fully prevented not only the enhancing effects of CSDS on mice immobility in the FST [ANOVA: CSDS, F(1, 36) = 53.350, *p* < 0.01; Vortioxetine, F(1, 36) = 23.576, *p* < 0.01; Interaction, F(1, 36) = 0.950, *p* = 0.336] and TST [ANOVA: CSDS, F(1, 36) = 37.330, *p* < 0.01; Vortioxetine, F(1, 36) = 25.741, *p* < 0.01; Interaction, F(1, 36) = 0.015, *p* = 0.903] but also the down-regulating effects of CSDS on the sucrose preference [ANOVA: CSDS, F(1, 36) = 10.456, *p* < 0.01; Vortioxetine, F(1, 36) = 4.661, *p* < 0.05; Interaction, F(1, 36) = 1.348, *p* = 0.253] and social interaction [ANOVA: CSDS, F(1, 36) = 84.394, *p* < 0.01; Vortioxetine, F(1, 36) = 30.286, *p* < 0.01; Interaction, F(1, 36) = 17.363, *p* < 0.01] of mice (*n* = 10). Thus, the antidepressant actions of vortioxetine were confirmed in mice.

Then, we did western blotting to evaluate the effects of vortioxetine on the protein expression of hippocampal PPARα. The results are displayed in Figure 1D and Figure 2E. Figure 1D indicates that while the protein level of hippocampal PPARα was down-regulated in the CUMS group compared to the vehicle-treated control group by 55.5 ± 7.18%, repeated vortioxetine injection notably increased it in the CUMS-exposed mice by 136.2 ± 11.64% [ANOVA: CUMS, F(1, 16) = 21.620, *p* < 0.01; Vortioxetine, F(1, 16) = 32.494, *p* < 0.01; Interaction, F(1, 16) = 38.035, *p* < 0.01] (*n* = 5). Similarly, Figure 2E shows that vortioxetine treatment fully prevented the reducing effects of CSDS [ANOVA: CSDS, F(1, 16) = 32.727, *p* < 0.01; Vortioxetine, F(1, 16) = 23.372, *p* < 0.01; Interaction, F(1, 16) = 12.285, *p* < 0.01] on the level of PPARα protein in the hippocampus (*n* = 5). Moreover, vortioxetine administration produced none effects on the hippocampal PPARα expression in naive control mice (*n* = 5). The protein expression of hippocampal β-actin was nearly the same between all groups (*n* = 5).

Taken together, the antidepressant actions of vortioxetine in rodent models may involve PPARα in the hippocampus.

### Pharmacological Inhibition of PPARα Attenuated the Protecting Effects of Vortioxetine Against Both Chronic Unpredictable Mild Stress and Chronic Social Defeat Stress

Then, GW6471 and MK886, two selective antagonists of PPARα, were adopted to investigate whether the antidepressant effects of vortioxetine in mice require PPARα. Therefore, mice subjected to chronic stress were co-treated with vortioxetine and PPARα antagonists for 2 weeks. Afterwards, behavioral tests and western blotting detection were performed.

**FIGURE 3:**
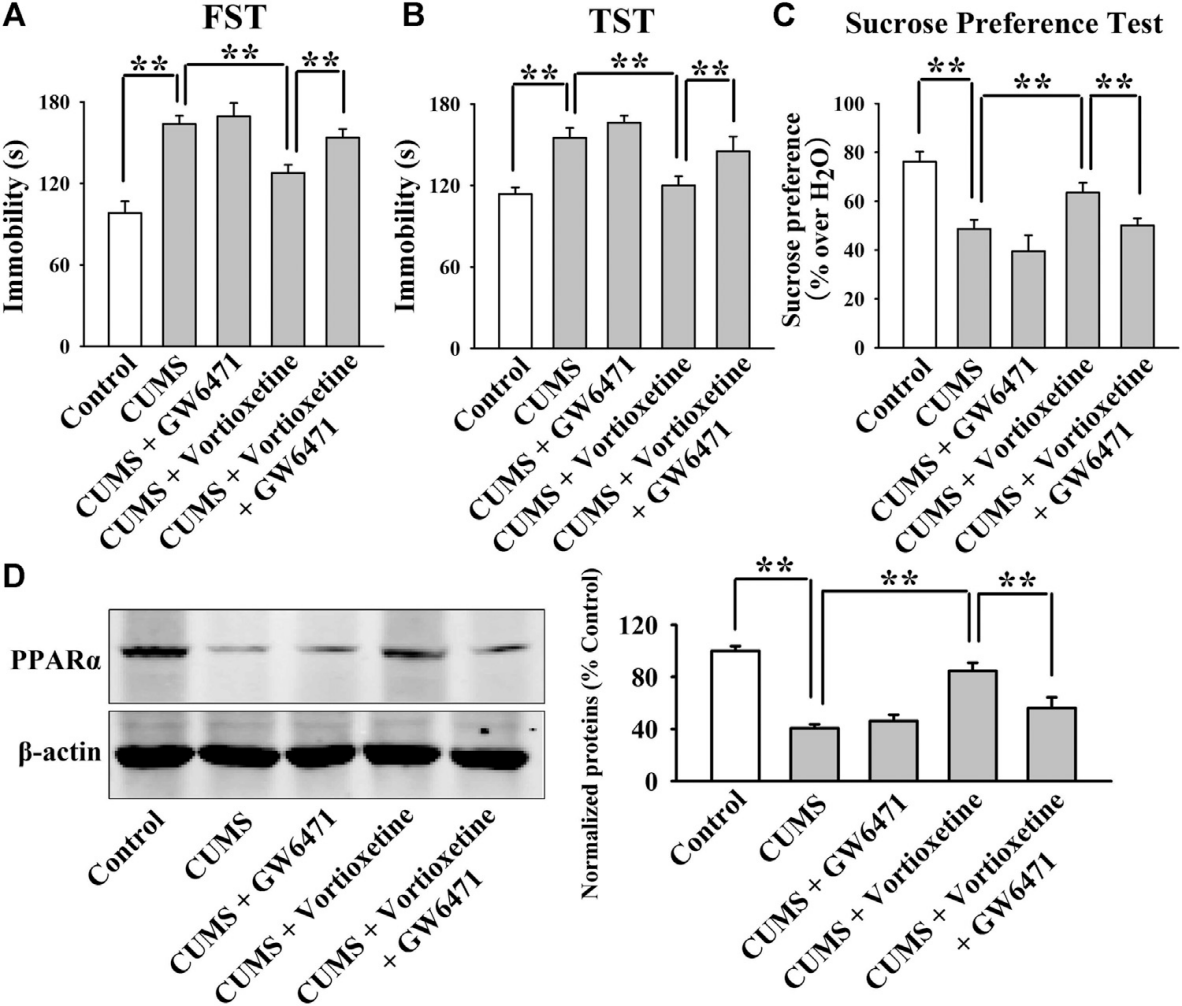
The antidepressant actions of vortioxetine in the CUMS model were fully blocked by GW6471 co-administration. **(A–C)** The reversal effects of vortioxetine on the CUMS-induced depressive-like behaviors in mice were fully blocked by GW6471 co-administration, as revealed by the FST, TST and sucrose preference test. (D) Representative images and quantitative analyses indicate that the use of GW6471 notably prevented the promoting effects of vortioxetine on the hippocampal PPARα expression in the CUMS-exposed mice. For **(A–C)**, *n* = 10 per group; For (D), *n* = 5 per group. The data are expressed as the means ± S.E.M.; **p < 0.01. The comparisons were made by two-way ANOVA followed by Bonferroni’s test.

**FIGURE 4:**
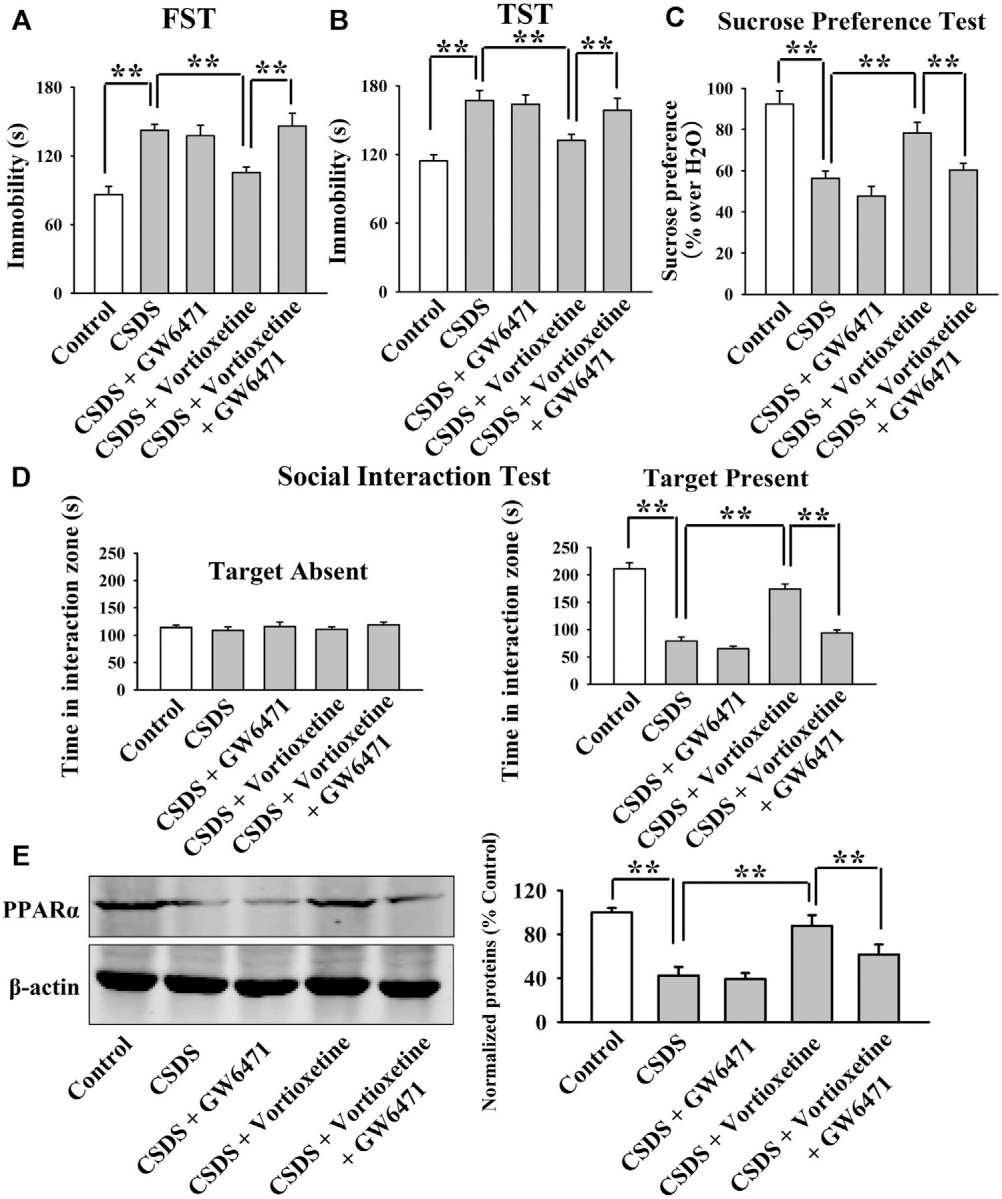
The antidepressant actions of vortioxetine in the CSDS model were fully blocked by GW6471 co-administration. **(A–D)** The reversal effects of vortioxetine on the CSDS-induced depressive-like behaviors in mice were fully blocked by GW6471 co-administration, as revealed by the FST, TST, sucrose preference test and social interaction test. **(E)** Representative images and quantitative analyses indicate that the use of GW6471 notably prevented the promoting effects of vortioxetine on the hippocampal PPARα expression in the CSDS-exposed mice. For **(A–D)**, *n* = 10 per group; For **(E)**, *n* = 5 per group. The data are expressed as the means ± S.E.M.; **p < 0.01. The comparisons were made by two-way ANOVA followed by Bonferroni’s test.

The GW6471 results are summarized in Figure 3 and Figure 4. As shown in Figures 3A–C, while vortioxetine administration prevented the depressive-like behaviors induced by CUMS in mice in the FST [ANOVA: GW6471, F(1, 36) = 4.981, *p* < 0.05; Vortioxetine, F(1, 36) = 12.951, *p* < 0.01; Interaction, F(1, 36) = 1.985, *p* = 0.167], TST [ANOVA: GW6471, F(1, 36) = 5.334, *p* < 0.05; Vortioxetine, F(1, 36) = 13.357, *p* < 0.01; Interaction, F(1, 36) = 0.812, *p* = 0.374] and sucrose preference test [ANOVA: GW6471, F(1, 36) = 5.964, *p* < 0.05; Vortioxetine, F(1, 36) = 7.970, *p* < 0.01; Interaction, F(1, 36) = 0.243, *p* = 0.625], these ameliorating effects were notably attenuated by GW6471 co-administration (*n* = 10). Similarly, Figures 4A–D reveal that while vortioxetine administration antagonized the depressive-like behaviors caused by CSDS in mice in the FST [ANOVA: GW6471, F(1, 36) = 5.212, *p* < 0.05; Vortioxetine, F(1, 36) = 8.723, *p* < 0.01; Interaction, F(1, 36) = 3.025, *p* = 0.091], TST [GW6471, F(1, 36) = 5.503, *p* < 0.05; Vortioxetine, F(1, 36) = 11.888, *p* < 0.01; Interaction, F(1, 36) = 3.349, *p* = 0.076], sucrose preference test [ANOVA: GW6471, F(1, 36) = 11.180, *p* < 0.01; Vortioxetine, F(1, 36) = 18.101, *p* < 0.01; Interaction, F(1, 36) = 1.408, *p* = 0.243] and social interaction test [ANOVA: GW6471, F(1, 36) = 43.563, *p* < 0.01; Vortioxetine, F(1, 36) = 77.075, *p* < 0.01; Interaction, F(1, 36) = 21.849, *p* < 0.01], these reversal actions were notably attenuated by GW6471 co-administration (*n* = 10). Meanwhile, Figure 3D and Figure 4E indicate that GW6471 co-administration notably blocked the protecting actions of vortioxetine against the inhibitory effects of both CUMS [ANOVA: GW6471, F(1, 16) = 8.085, *p* < 0.01; Vortioxetine, F(1, 16) = 20.484, *p* < 0.01; Interaction, F(1, 16) = 3.615, *p* = 0.075] and CSDS [ANOVA: GW6471, F(1, 16) = 7.975, *p* < 0.01; Vortioxetine, F(1, 16) = 17.568, *p* < 0.01; Interaction, F(1, 16) = 3.434, *p* = 0.082] on hippocampal PPARα (*n* = 5), consistent with the behavioral results.

**FIGURE 5:**
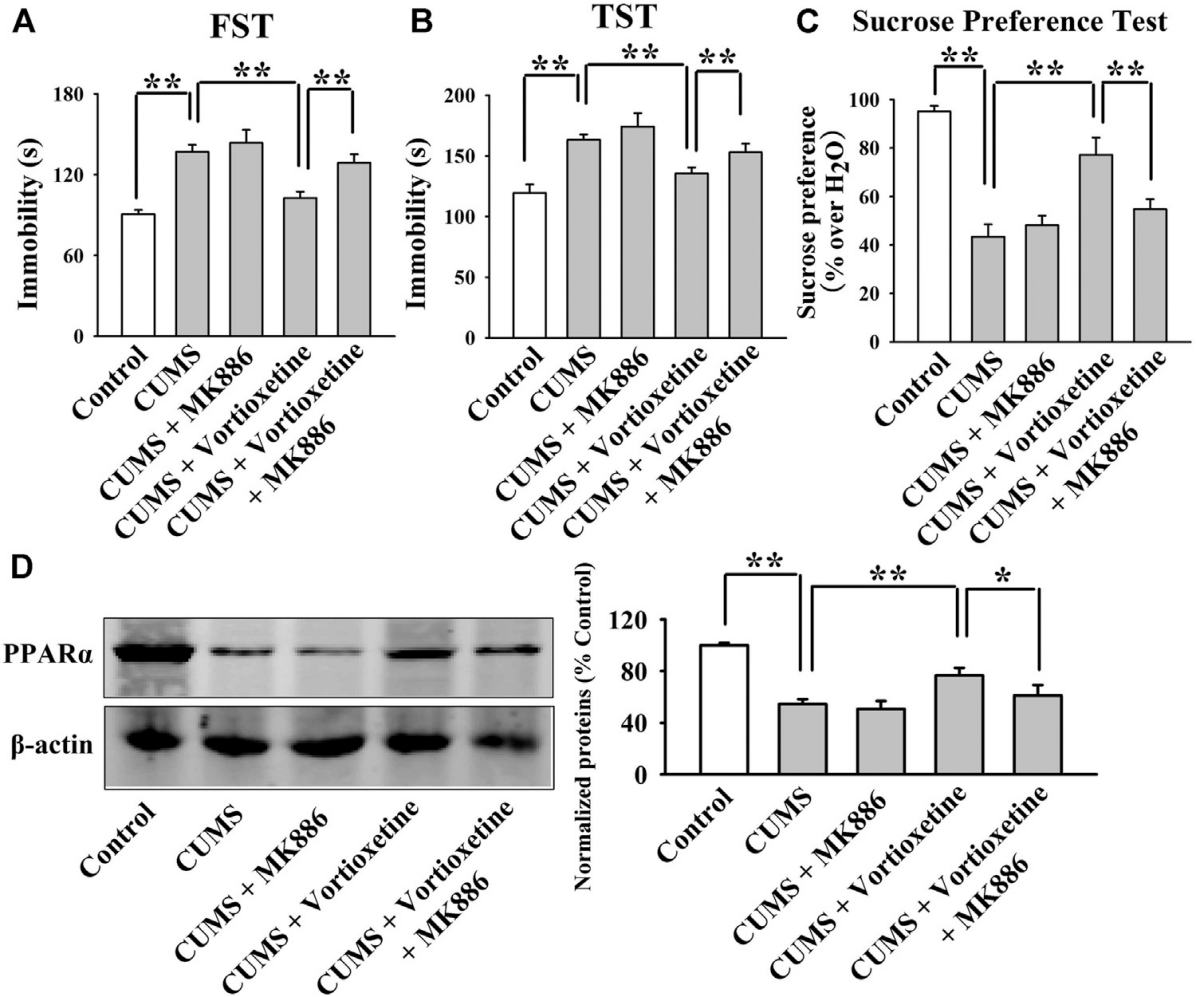
The antidepressant effects of vortioxetine in the CUMS model were significantly prevented by MK886 co-treatment. **(A–C)** The reversal effects of vortioxetine on the CUMS-induced depressive-like behaviors in mice were significantly prevented by MK886 co-treatment, as indicated by the FST, TST and sucrose preference test. **(D)** Representative images and quantitative analyses show that the use of MK886 notably blocked the enhancing effects of vortioxetine on the hippocampal PPARα expression in the CUMS-exposed mice. For **(A–C)**, *n* = 10 per group; For **(D)**, *n* = 5 per group. The data are expressed as the means ± S.E.M.; *p < 0.05, **p < 0.01. The comparisons were made by two-way ANOVA followed by Bonferroni’s test.

**FIGURE 6:**
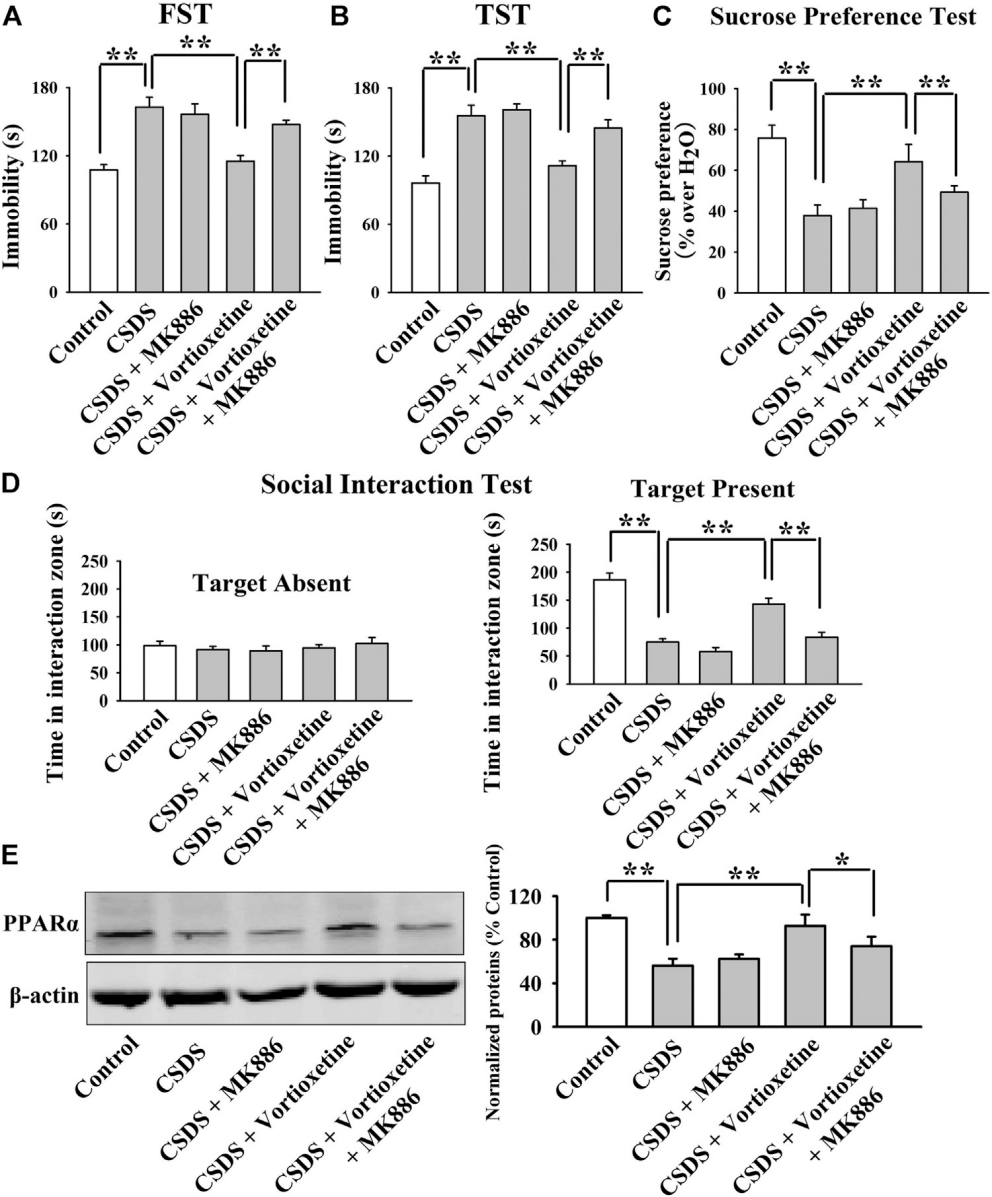
The antidepressant effects of vortioxetine in the CSDS model were significantly prevented by MK886 co-treatment. **(A–D)** The reversal effects of vortioxetine on the CSDS-induced depressive-like behaviors in mice were significantly prevented by MK886 co-treatment, as indicated by the FST, TST, sucrose preference test and social interaction test. **(E)** Representative images and quantitative analyses show that the use of MK886 notably blocked the enhancing effects of vortioxetine on the hippocampal PPARα expression in the CSDS-exposed mice. For **(A–D)**, *n* = 10 per group; For **(E)**, *n* = 5 per group. The data are expressed as the means ± S.E.M.; *p < 0.05, **p < 0.01. The comparisons were made by two-way ANOVA followed by Bonferroni’s test.

The MK886 results are summarized in Figure 5 and Figure 6. As shown in Figures 5A–C, while vortioxetine treatment prevented the depressive-like behaviors induced by CUMS in mice in the FST [ANOVA: MK886, F(1, 36) = 5.936, *p* < 0.05; Vortioxetine, F(1, 36) = 10.759, *p* < 0.01; Interaction, F(1, 36) = 3.225, *p* = 0.084], TST [MK886, F(1, 36) = 4.287, *p* < 0.05; Vortioxetine, F(1, 36) = 9.147, *p* < 0.01; Interaction, F(1, 36) = 1.231, *p* = 0.269] and sucrose preference test [ANOVA: MK886, F(1, 36) = 7.889, *p* < 0.01; Vortioxetine, F(1, 36) = 15.466, *p* < 0.01; Interaction, F(1, 36) = 4.413, *p* < 0.05], these ameliorating effects were notably blocked by MK886 co-treatment (*n* = 10). Also, Figures 6A–D reveal that while vortioxetine treatment antagonized the depressive-like behaviors caused by CSDS in mice in the FST [MK886, F(1, 36) = 7.953, *p* < 0.01; Vortioxetine, F(1, 36) = 11.059, *p* < 0.01; Interaction, F(1, 36) = 1.922, *p* = 0.171], TST [ANOVA: MK886, F(1, 36) = 8.915, *p* < 0.01; Vortioxetine, F(1, 36) = 14.716, *p* < 0.01; Interaction, F(1, 36) = 2.774, *p* = 0.124], sucrose preference test [ANOVA: MK886, F(1, 36) = 5.109, *p* < 0.05; Vortioxetine, F(1, 36) = 10.436, *p* < 0.01; Interaction, F(1, 36) = 1.365, *p* = 0.251] and social interaction test [ANOVA: MK886, F(1, 36) = 34.189, *p* < 0.01; Vortioxetine, F(1, 36) = 57.816, *p* < 0.01; Interaction, F(1, 36) = 17.088, *p* < 0.01], these reversal actions were notably blocked by MK886 co-treatment (*n* = 10). Moreover, Figure 5D and Figure 6E indicate that MK886 co-administration notably blocked the protecting actions of vortioxetine against the inhibitory effects of both CUMS [ANOVA: MK886, F(1, 16) = 7.198, *p* < 0.05; Vortioxetine, F(1, 16) = 7.156, *p* < 0.05; Interaction, F(1, 16) = 0.757, *p* = 0.397] and CSDS [ANOVA: MK886, F(1, 16) = 8.564, *p* < 0.01; Vortioxetine, F(1, 16) = 9.532, *p* < 0.01; Interaction, F(1, 16) = 0.651, *p* = 0.431] on hippocampal PPARα(*n* = 5), consistent with the behavioral results.

Collectively, pharmacological inhibition of PPARα attenuated the protecting effects of vortioxetine against the CUMS and CSDS models of depression.

### Genetic Knockdown of Hippocampal PPARα Abolished the Protecting Effects of Vortioxetine Against Both Chronic Unpredictable Mild Stress and Chronic Social Defeat Stress

Furthermore, AAV-PPARα-shRNA was used to selectively knockdown PPARα expression. The silencing efficacy of AAV-PPARα-shRNA has been demonstrated in Supplementary Figure S1 and our previous study (Song et al., 2018). In brief, the mice stereotactically infused with AAV-PPARα-shRNA were maintained for 2 weeks and then subjected to chronic stress and vortioxetine treatment. Afterwards, behavioral tests and western blotting detection were performed.

**FIGURE 7:**
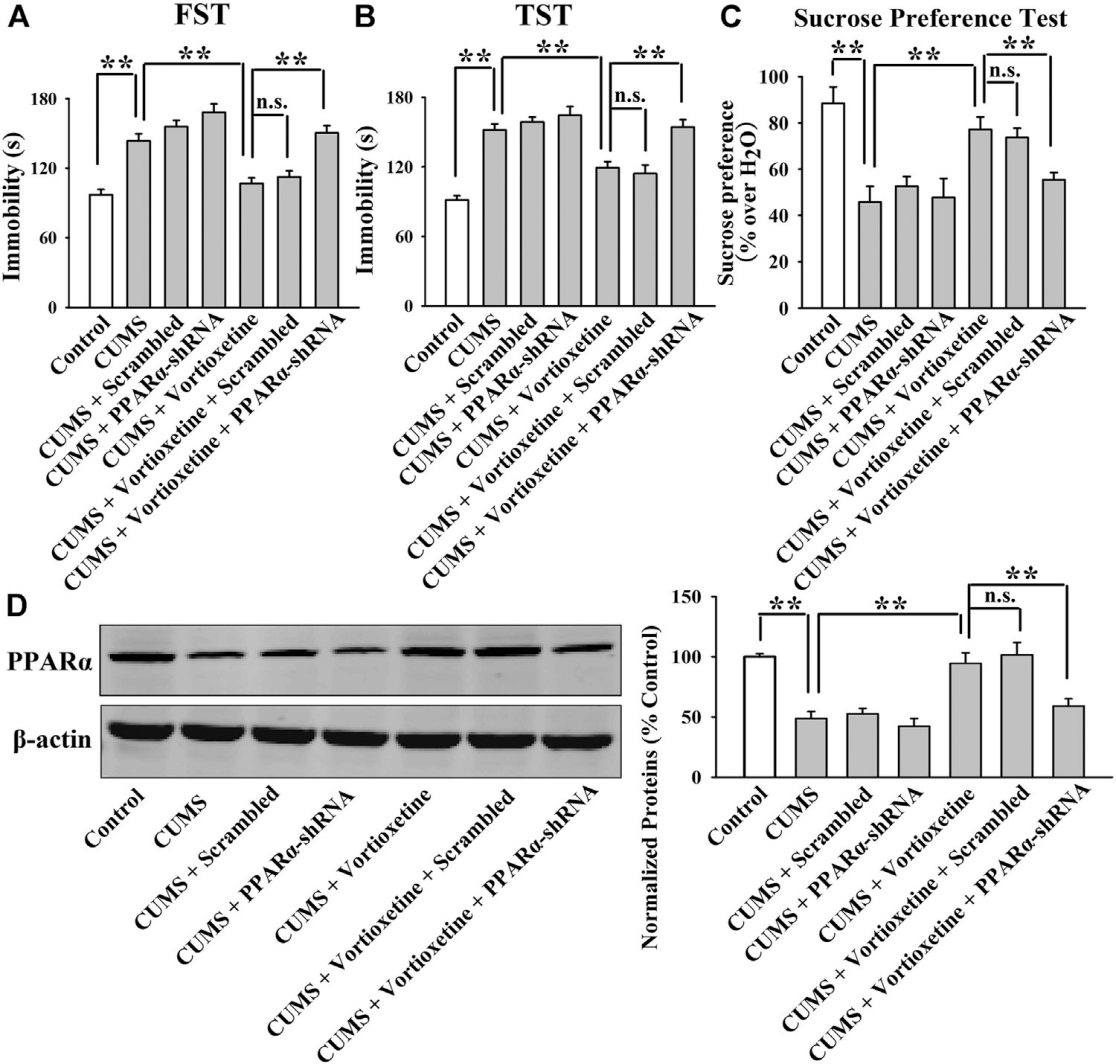
AAV-PPARα-shRNA completely abolished the antidepressant-like effects of vortioxetine in the CUMS model. **(A–C)** The (CUMS + vortioxetine + PPARα-shRNA)-treated mice exhibited significantly longer immobility in the FST and TST as well as lower sucrose preference than the (CUMS + vortioxetine)-treated and (CUMS + vortioxetine + Scrambled)-treated mice. (D) The (CUMS + vortioxetine + PPARα-shRNA)-treated mice displayed notably less expression of hippocampal PPARα than the (CUMS + vortioxetine)-treated and (CUMS + vortioxetine + Scrambled)-treated mice. For **(A–C)**, n = 10 per group; For **(D)**, *n* = 5 per group. The data are expressed as the means ± S.E.M.; **p < 0.01; n.s., no significance. The comparisons were made by two-way ANOVA followed by Bonferroni’s test.

**FIGURE 8:**
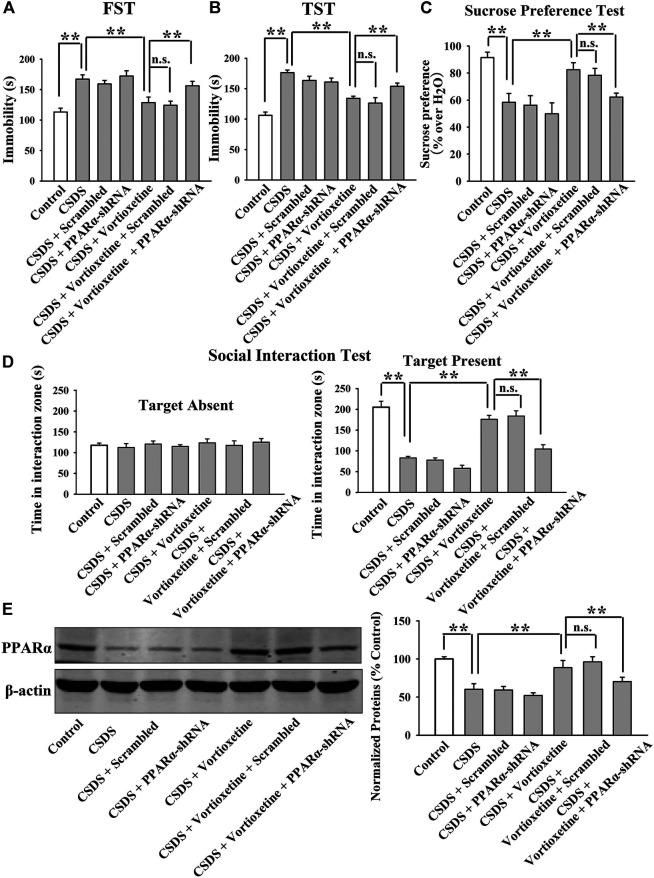
AAV-PPARα-shRNA completely abolished the antidepressant-like actions of vortioxetine in the CSDS model. **(A–D)** The (CSDS + vortioxetine + PPARα-shRNA)-treated mice showed significantly longer immobility in the FST and TST as well as lower sucrose preference and social interaction than the (CSDS + vortioxetine)-treated and (CSDS + vortioxetine + Scrambled)-treated mice. **(D)** The (CSDS + vortioxetine + PPARα-shRNA)-treated mice had notably less expression of hippocampal PPARα than the (CSDS + vortioxetine)-treated and (CSDS + vortioxetine + Scrambled)-treated mice. For **(A–D)**, *n* = 10 per group; For **(E)**, *n* = 5 per group. The data are expressed as the means ± S.E.M.; **p < 0.01; n. s., no significance. The comparisons were made by two-way ANOVA followed by Bonferroni’s test.

The results of the CUMS experiments are displayed in Figure 7. It was found that genetic knockdown of hippocampal PPARα significantly prevented the decrease induced by vortioxetine treatment in the immobility of mice exposed to CUMS in the FST [ANOVA: PPARα-shRNA, F(1, 36) = 29.956, *p* < 0.01; Vortioxetine, F(1, 36) = 19.936, *p* < 0.01; Interaction, F(1, 36) = 2.410, *p* = 0.129; Figure 7A] and TST [ANOVA: PPARα-shRNA, F(1, 36) = 15.298, *p* < 0.01; Vortioxetine, F(1, 36) = 11.903, *p* < 0.01; Interaction, F(1, 36) = 3.494, *p* = 0.070; Figure 7B] (*n* = 10). Additionally, genetic knockdown of hippocampal PPARα significantly prevented the increase induced by vortioxetine administration in the sucrose preference of mice exposed to CUMS [ANOVA: PPARα-shRNA, F(1, 36) = 4.187, *p* < 0.05; Vortioxetine, F(1, 36) = 10.729, *p* < 0.01; Interaction, F(1, 36) = 2.749, *p* = 0.106; Figure 7C] (*n* = 10). The results of the CSDS experiments are displayed in Figure 8. It was found that genetic knockdown of hippocampal PPARα fully blocked the protecting effects of vortioxetine against the CSDS-induced depressive-like behaviors in the FST [ANOVA: PPARα-shRNA, F(1, 36) = 4.755, *p* < 0.05; Vortioxetine, F(1, 36) = 11.481, *p* < 0.01; Interaction, F(1, 36) = 1.877, *p* = 0.179; Figure 8A], TST [ANOVA: PPARα-shRNA, F(1, 36) = 11.778, *p* < 0.01; Vortioxetine, F(1, 36) = 22.099, *p* < 0.01; Interaction, F(1, 36) = 0.127, *p* = 0.724; Figure 8B], sucrose preference test [ANOVA: PPARα-shRNA, F(1, 36) = 6.182, *p* < 0.05; Vortioxetine, F(1, 36) = 9.843, *p* < 0.01; Interaction, F(1, 36) = 0.842, *p* = 0.365; Figure 8C] and social interaction test [ANOVA: PPARα-shRNA, F(1, 36) = 35.772, *p* < 0.01; Vortioxetine, F(1, 36) = 77.141, *p* < 0.01; Interaction, F(1, 36) = 9.246, *p* < 0.01; Figure 8D] (*n* = 10). In contrast, scrambled control-shRNA produced none influence in the behavioral tests. Moreover, PPARα-shRNA co-administration notably abolished the reversal effects of vortioxetine on the down-regulated PPARα expression in the hippocampus of both the CUMS-exposed [ANOVA: PPARα-shRNA, F(1, 16) = 9.037, *p* < 0.01; Vortioxetine, F(1, 16) = 21.211, *p* < 0.01; Interaction, F(1, 16) = 4.739, *p* < 0.05] and CSDS-exposed [ANOVA: PPARα-shRNA, F(1, 16) = 7.718, *p* < 0.05; Vortioxetine, F(1, 16) = 11.022, *p* < 0.01; Interaction, F(1, 16) = 0.505, *p* = 0.488] mice (*n* = 5), consistent with the behavioral results.

In summary, combined with the above results involving PPARα antagonists, it can be concluded that the antidepressant mechanism of vortioxetine require hippocampal PPARα.

## Discussion

So far there are a variety of available pharmacological options in clinical practice for treating MDD, mainly including monoamine oxidase inhibitors (MAOIs), tricyclic antidepressants (TCAs), SSRIs, SNRIs and some other antidepressants (e.g. mirtazapine, agomelatine, reboxetine). Current guidelines for depression recommend SSRIs over SNRIs, MAOIs and TCAs due to their favorable adverse effect profile (Bauer et al., 2007; Cleare et al., 2015). SSRIs have now become the most prescribed antidepressant class in most parts of the world. Vortioxetine was licensed for treating MDD in 2013. Despite the similarities to SSRIs, the pharmacological mechanism of vortioxetine is claimed to be novel (Koesters et al., 2017). Vortioxetine is now placed in the category of “Other” antidepressants according to the ATC classification of the World Health Organization (Koesters et al., 2017).

Although proved to be an antagonist at 5-HT_3_, 5-HT_7_ and 5-HT_1D_, a partial agonist at 5-HT_1B_, an agonist at 5-HT_1A_ and an inhibitor of SERT, the antidepressant mechanism of vortioxetine is actually not fully understood. It is unclear if and how these modulating actions of vortioxetine on the 5-HT receptors and SERT contribute to its antidepressant effects (Koesters et al., 2017). There is a hypothesis describing that the effects of vortioxetine on the 5-HT receptors and SERT lead to enhanced release of 5-HT, NA, dopamine, histamine, acetylcholine and glutamate as well as decreased release of aminobutyric acid (GABA), which then improve the efficiency of information processing in malfunctioning brain circuits by facilitating long-term potentiation (LTP), neuroplasticity and firing of pyramidal neurons (Koesters et al., 2017). Here, our study is the first comprehensive *in vivo* evidence suggesting that vortioxetine induces antidepressant effects in mice models of depression by significantly promoting the hippocampal PPARα expression. Our study extends the knowledge of vortioxetine’s pharmacological effects and further highlights the role of PPARα in depression.

Due to the recent marketing authorization, molecular studies on the pharmacological actions of vortioxetine are limited until now. For example, Yu *et al.*, Lu *et al.* and Sun *et al*., (Yu et al., 2017; Lu et al., 2018; Sun et al., 2020) all reported that vortioxetine administration significantly increased the hippocampal brain-derived neurotrophic factor (BDNF) -cAMP-response element binding protein (CREB) signaling cascade in rodent models of depression. Du Jardin *et al*., (du Jardin et al., 2016) showed that a single dose of vortoxetine increased the expression of several plasticity-related genes (mTOR, Mglur1, Pkcα, Homer3, Spinophilin, and Synapsin3) in the rat frontal cortex. Waller *et al*., (Waller et al., 2017) indicated that chronic administration of vortioxetine in rodents modulated several neurodevelopmental and plasticity markers such as Sema4g. Kugathasan *et al.,* (Kugathasan et al., 2017) demonstrated that vortioxetine has promoting effects on the function of molecules associated with neuroplasticity which include Arc/Arg3.1, GluA1 and Ca^2+^/calmodulin-dependent kinase α. Moreover, BDNF is critical for LTP, neuroplasticity and neuronal activity (Lu et al., 2008; Leal et al., 2015; Graves et al., 2016). From these literatures and the hypothesis described above it can be seen that BDNF and neuroplasticity shall play important roles in the antidepressant response to vortioxetine treatment. How does vortioxetine affect BDNF and neuroplasticity? Here, our study provides a candidate, PPARα, as this protein not only correlates with BDNF biosynthesis and neuroplasticity but also is implicated in the pathogenesis of depression. Roy *et al.*, (Roy et al., 2013; Roy et al., 2015) reported that PPARα modulated the expression of several neuroplasticity-related proteins including BDNF via regulating the transcriptional activity of CREB. There have been several studies regarding the role of hippocampal PPARα in depression neurobiology and antidepressant responses, including ours and others. We have demonstrated that several PPARα agonists (WY14643, fenofibrate and gemfibrozil) all produced notable antidepressant-like effects in mice through activation of the hippocampal BDNF system, and that genetic regulation of hippocampal PPARα rendered mice susceptible/resilient to chronic stress (Jiang et al., 2015; Jiang et al., 2017; Ni et al., 2018; Song et al., 2018). We have also found that the antidepressant mechanisms of fluoxetine involve hippocampal PPARα (Song et al., 2018). In addition, the HMG-CoA reductase inhibitor, simvastatin, is reported to protect against CUMS in rats by enhancing the level of PPARα-CREB-BDNF pathway in the hippocampus (Roy et al., 2015). However, a report in 2015 mentioned that repeated injection of ketamine, a well-known fast-acting antidepressant, down-regulated the expression of cortical PPARα in wild type mice, supporting an opposite opinion from our studies (D'Agostino et al., 2015).

As to by which way vortioxetine increases the expression of hippocampal PPARα, one possibility is that like WY14643, fenofibrate and gemfibrozil, vortioxetine directly binds and activates PPARα, functioning as a PPARα agonist. To validate this possibility, further in-depth studies involving time-resolved FRET, electrospray ionization MS and in silico structural analysis are needed (Roy et al., 2013). It is also possible that the 5-HT-mediated signaling pathway underlies the effects of vortioxetine on hippocampal PPARα. However, there are no studies directly showing the correlation between PPARα and any 5-HT-mediated signaling pathway until now. Since PPARα is also implicated in the pathophysiology of several other neurological disorders (e.g. stroke, Alzheimer’s disease, Parkinson’s disease and epilepsy) besides depression (Bordet et al., 2006; Fidaleo et al., 2014; D'Orio et al., 2018), vortioxetine may possess more beneficial efficacy involving PPARα in the central nervous system. In the peripheral system, vortioxetine may have protective effects against atherosclerosis by influencing hepatic PPARα (Yu et al., 2015), like the fibrates. All these assumptions are very interesting and meaningful and deserve further investigation. Moreover, this study may have some limitations/deficiencies. For example, some previous reports suggested that the FST and TST could not evaluate “desperate state” or depressive-like behaviors, but refer more to coping behavior or learning in animals (Molendijk and de Kloet, 2015; de Kloet and Molendijk, 2016). Krishnan *et al.* showed that CSDS did not influence mice behaviors in the FST and TST, in contrary to our results (Krishnan et al., 2007). Another shortage of this study is using only rodent models, and its conclusion can be strongly strengthened if human tissue samples are involved.

In addition to PPARα, there are a lot of other proteins that not only control BDNF biosynthesis and neuroplasticity but also are implicated in depression, such as salt-inducible kinase 2, glycogen synthase kinase 3β and mammalian target of rapamycin (Zhou et al., 2014; Peng et al., 2018; Jiang et al., 2019). These proteins may also play a role in the antidepressant actions of vortioxetine, and more extensive research will be exhibited in the future.

## Data Availability

The original contributions presented in the study are included in the article/Supplementary Material, further inquiries can be directed to the corresponding authors.

## Abbreviations

AAV, adenovirus associated virus; ANOVA, analysis of variance; BDNF, brain-derived neurotrophic factor; CREB, cAMP response element binding protein; CSDS, chronic social defeat stress; CUMS, chronic unpredictable mild stress; FST, forced swim test; 5-HT, serotonin; LTP, long-term potentiation; MAOI, monoamine oxidase inhibitor; MDD, major depressive disorder; PPARα, peroxisome proliferator activated receptor α; SERT, serotonin transporter; shRNA, short hairpin RNA; SNRI, serotonin and norepinephrine reuptake inhibitor; SSRI, selective serotonin-reuptake inhibitor; TCA, tricyclic antidepressant; TST, tail suspension test.
