# Supplementary figures and images for: Hippocampal PPARα Plays a Role in the Pharmacological Mechanism of Vortioxetine, a Multimodal-Acting Antidepressant
Source: Front Pharmacol. 2021 Jun 15;12:673221. doi: 10.3389/fphar.2021.673221 (PMC8239178; doi:10.3389/fphar.2021.673221)

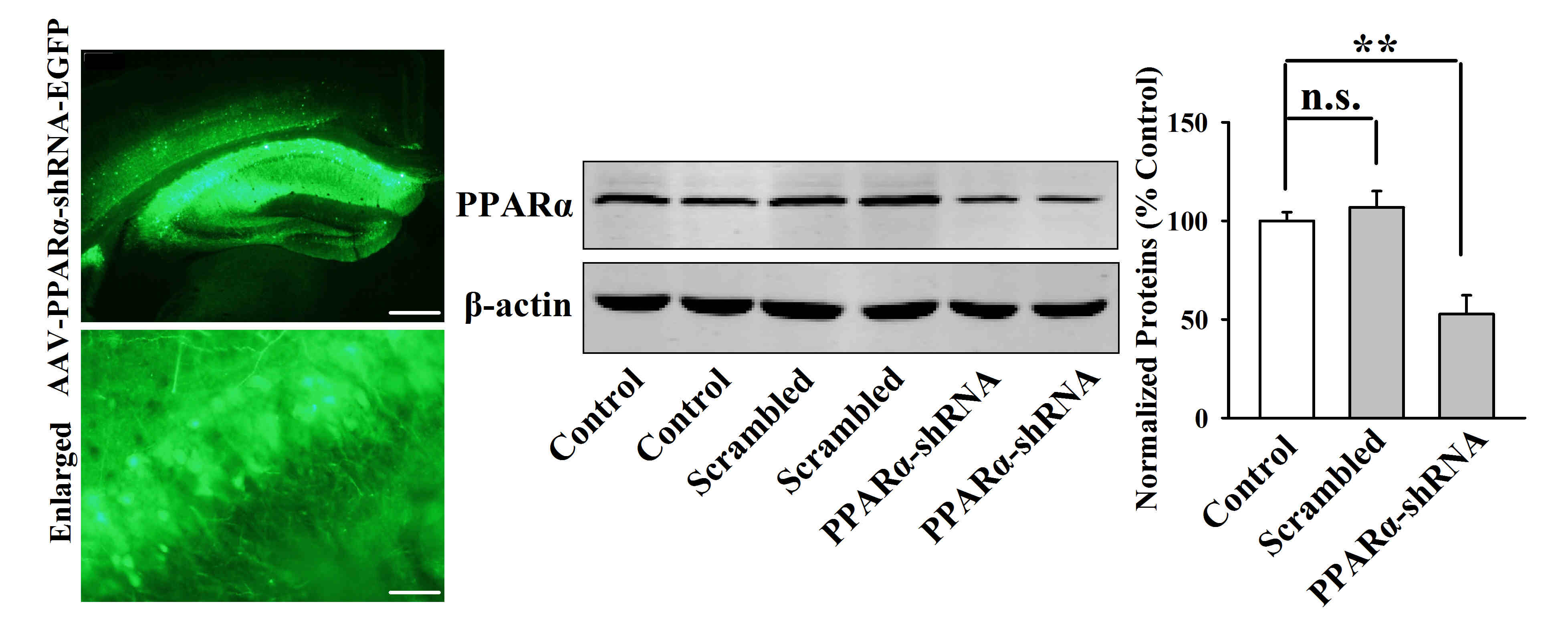

Supplement: Supplementary file 1 [file Image1.JPEG]
