# Supplementary material for: Hippocampal PPARα Plays a Role in the Pharmacological Mechanism of Vortioxetine, a Multimodal-Acting Antidepressant
Source: Front Pharmacol. 2021 Jun 15;12:673221. doi: 10.3389/fphar.2021.673221 (PMC8239178; doi:10.3389/fphar.2021.673221)
Supplement: Supplementary file 2 [file DataSheet1.PDF]

Supplemental Information

Supplemental Figure Legends

Figure S1. AAV-PPARα-shRNA-EGFP showed stable expression in the hippocampus region 2 weeks after stereotactic injection. The scale bar is 200 μm for the representative image and 25 μm for the enlarged image. Western blotting analysis confirmed the silencing efficacy of PPARα-shRNA (n = 5). The data are expressed as the means ± S.E.M.; ***P*< 0.01; n.s., no significance. The comparisons were made by one-way ANOVA followed by post-hoc Tukey's test.

The uncropped images for all western blots

Figure 1D

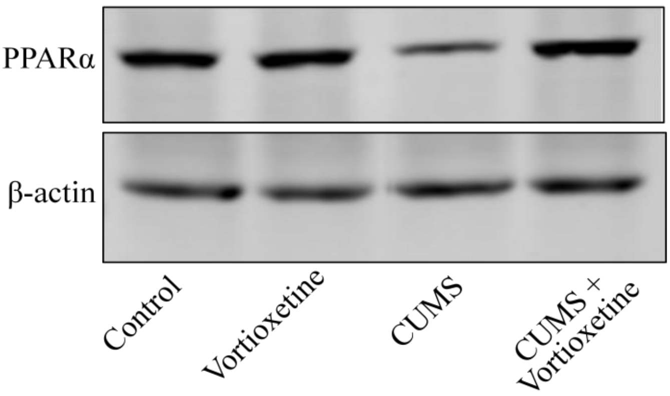

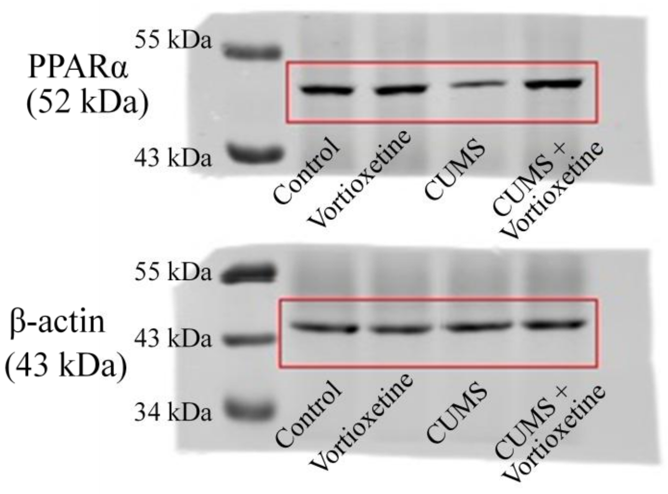

Figure 2E

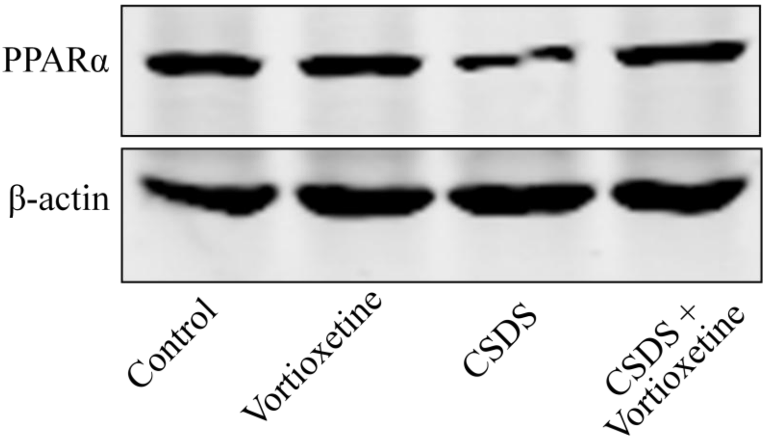

PPARα
β-actin
Control
Vortioxetine
CSDS
CSDS + Vortioxetine

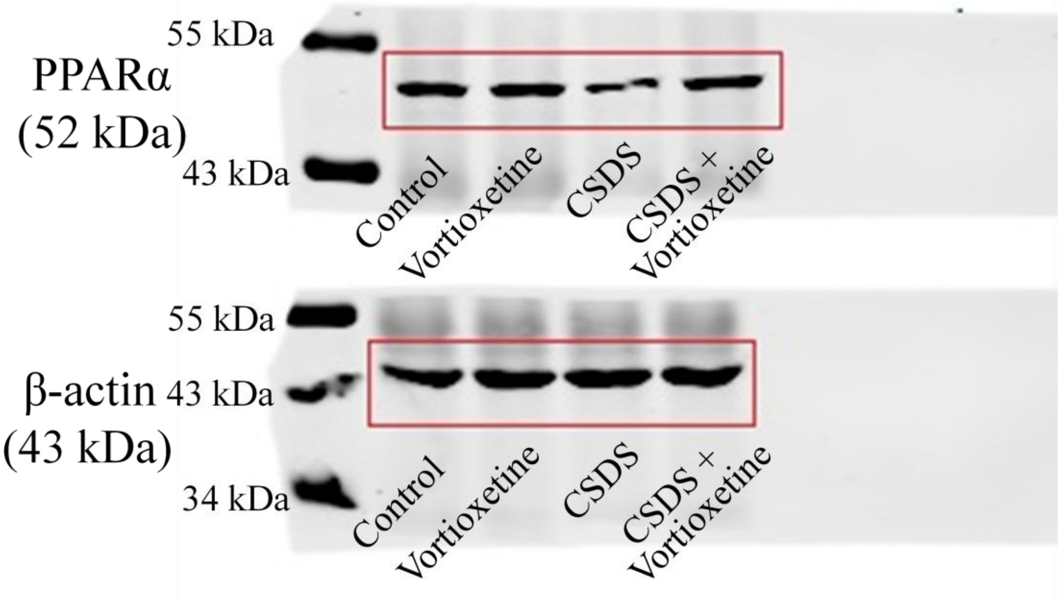

55 kDa
PPARα
(52 kDa)
43 kDa
Control
Vortioxetine
CSDS
CSDS + Vortioxetine
55 kDa
β-actin
(43 kDa)
43 kDa
34 kDa
Control
Vortioxetine
CSDS
CSDS + Vortioxetine

Figure 3D

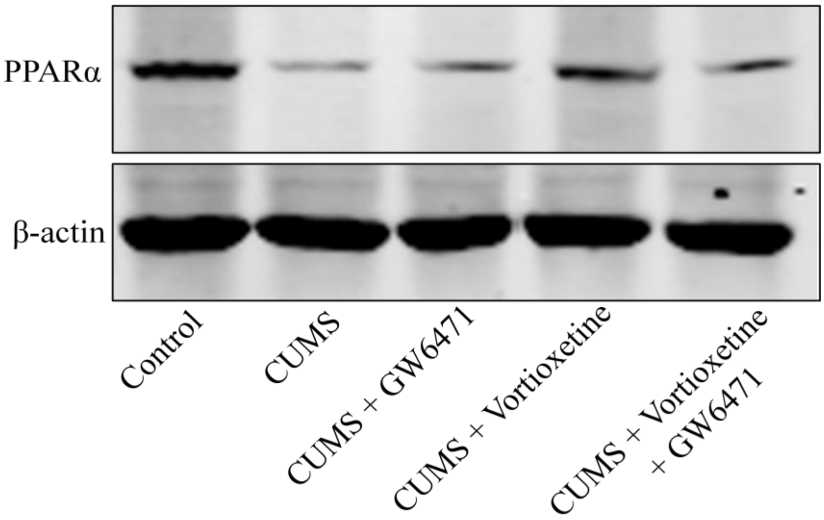
PPARα
β-actin
Control
CUMS
CUMS + GW6471
CUMS + Vortioxetine
CUMS + Vortioxetine + GW6471

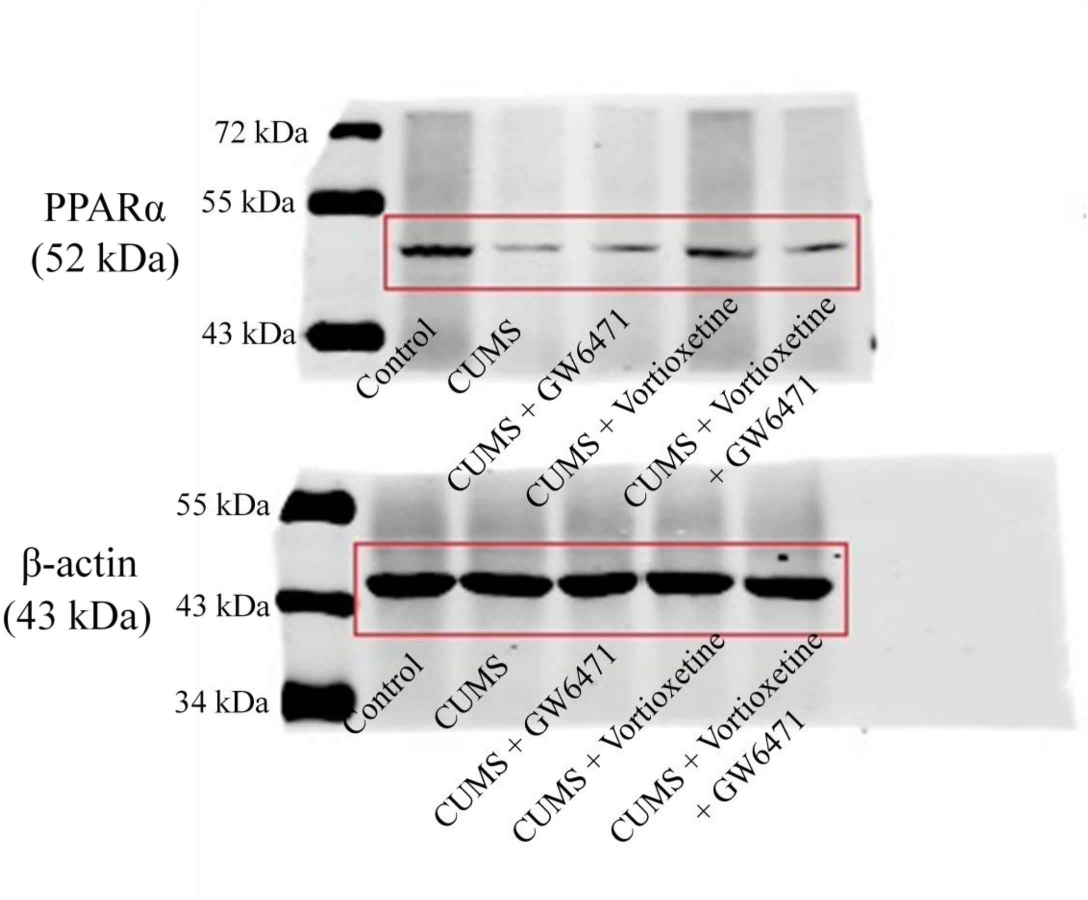
PPARα (52 kDa)
72 kDa
55 kDa
43 kDa
Control
CUMS
CUMS + GW6471
CUMS + Vortioxetine
CUMS + Vortioxetine + GW6471
β-actin (43 kDa)
55 kDa
43 kDa
34 kDa
Control
CUMS
CUMS + GW6471
CUMS + Vortioxetine
CUMS + Vortioxetine + GW6471

Figure 4E

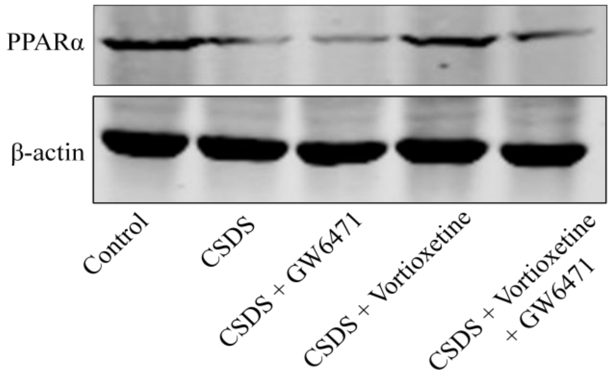
PPARα
β-actin
Control
CSDS
CSDS + GW6471
CSDS + Vortioxetine
CSDS + Vortioxetine + GW6471

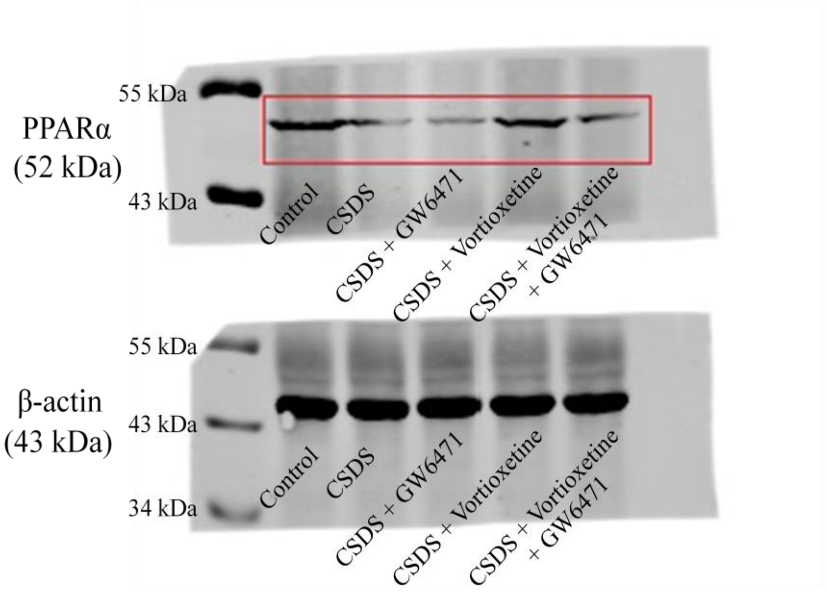
55 kDa
PPARα (52 kDa)
43 kDa
Control
CSDS
CSDS + GW6471
CSDS + Vortioxetine
CSDS + Vortioxetine + GW6471
55 kDa
β-actin (43 kDa)
43 kDa
34 kDa
Control
CSDS
CSDS + GW6471
CSDS + Vortioxetine
CSDS + Vortioxetine + GW6471

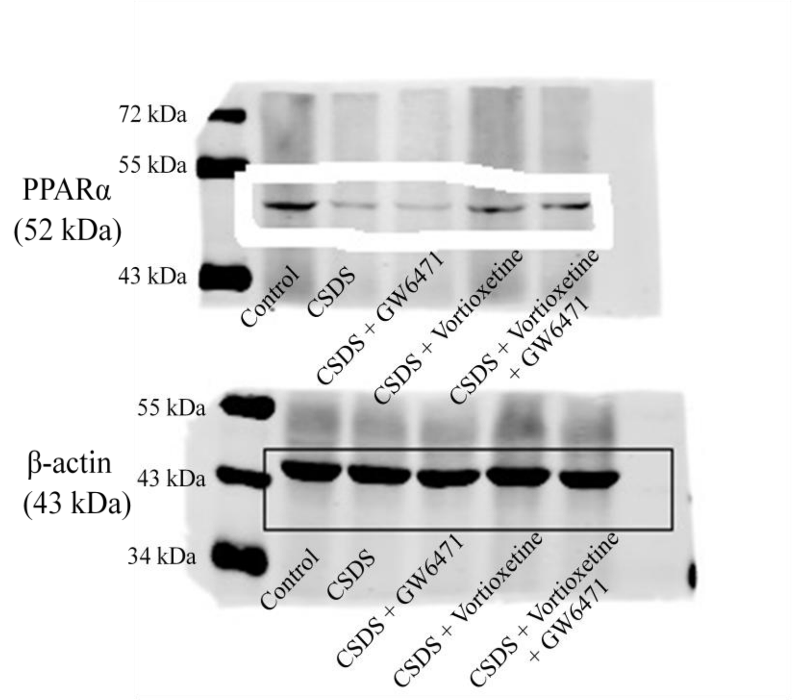
72 kDa
55 kDa
PPARα (52 kDa)
43 kDa
Control
CSDS
CSDS + GW6471
CSDS + Vortioxetine
CSDS + Vortioxetine + GW6471
55 kDa
β-actin (43 kDa)
43 kDa
34 kDa
Control
CSDS
CSDS + GW6471
CSDS + Vortioxetine
CSDS + Vortioxetine + GW6471

Figure 5D

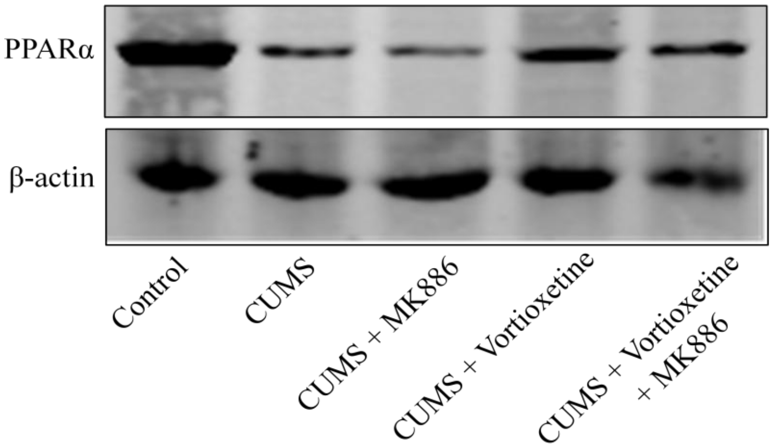
PPARα
β-actin
Control
CUMS
CUMS + MK886
CUMS + Vortioxetine
CUMS + Vortioxetine + MK886

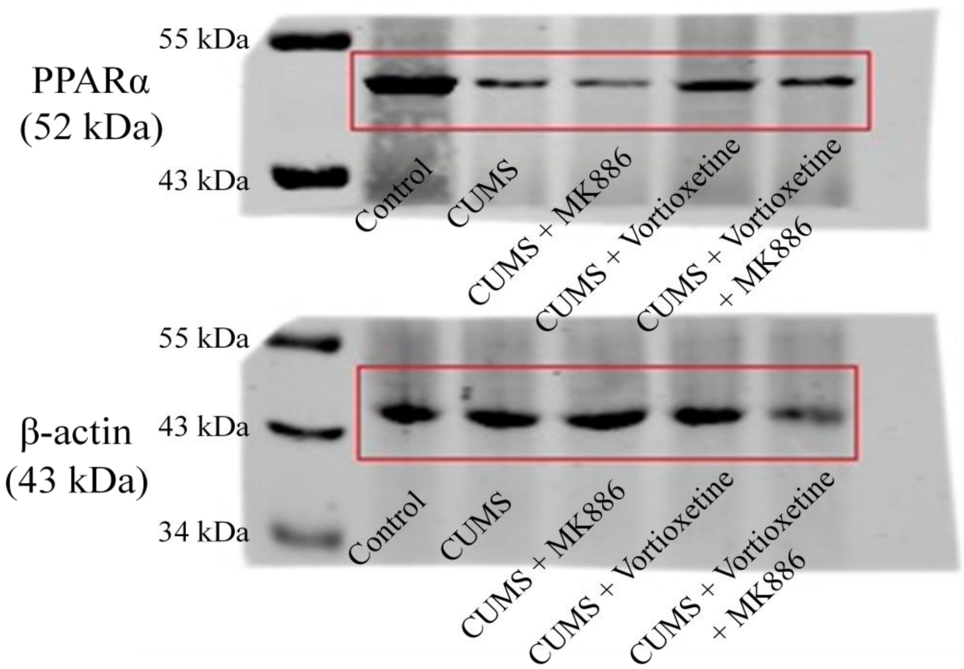
55 kDa
PPARα (52 kDa)
43 kDa
Control
CUMS
CUMS + MK886
CUMS + Vortioxetine
CUMS + Vortioxetine + MK886
55 kDa
β-actin (43 kDa)
43 kDa
34 kDa
Control
CUMS
CUMS + MK886
CUMS + Vortioxetine
CUMS + Vortioxetine + MK886

Figure 7D

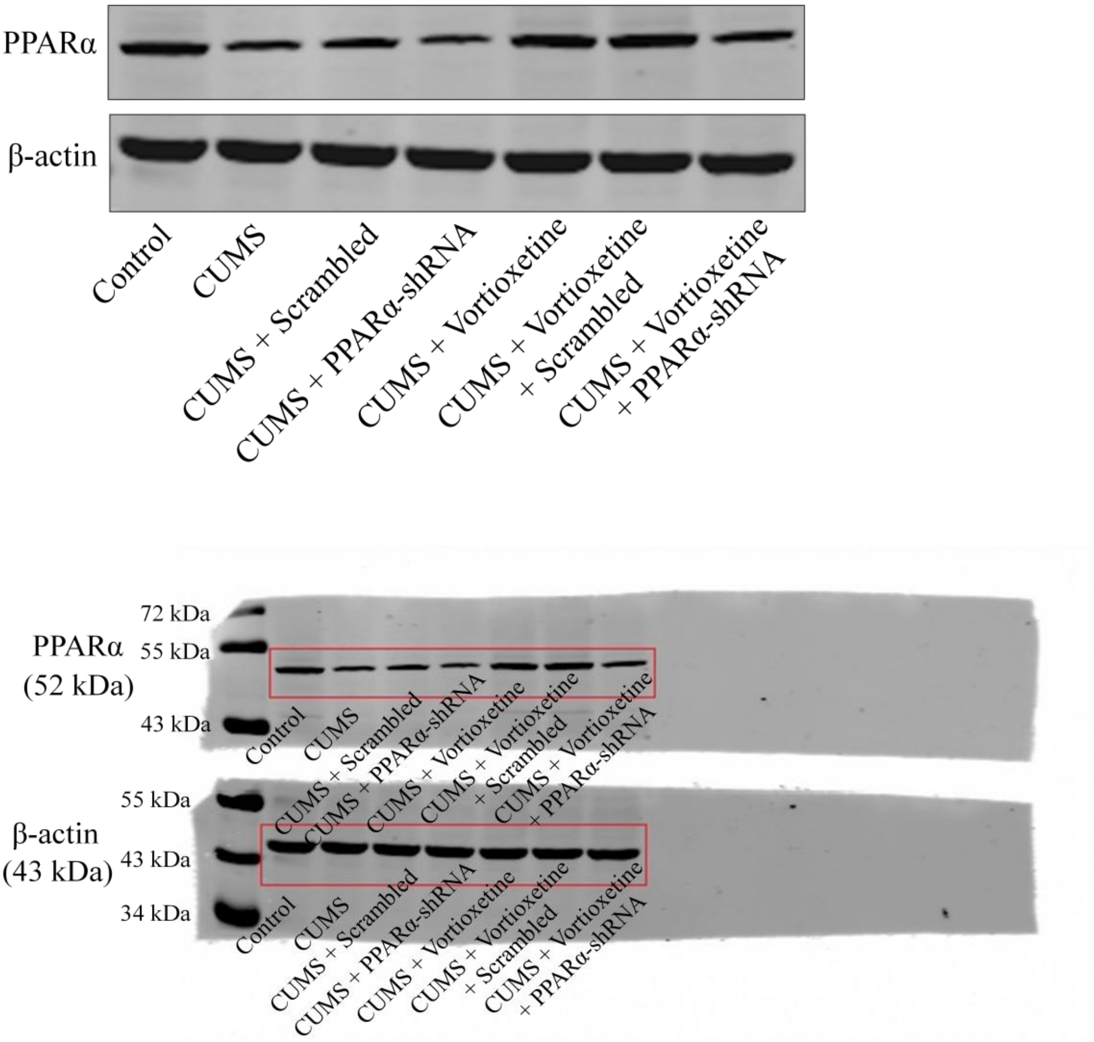
PPARα
β-actin
Control
CUMS
CUMS + Scrambled
CUMS + PPARα-shRNA
CUMS + Vortioxetine
CUMS + Vortioxetine + Scrambled
CUMS + Vortioxetine + PPARα-shRNA
PPARα (52 kDa)
β-actin (43 kDa)
72 kDa
55 kDa
43 kDa
34 kDa

Figure 8E

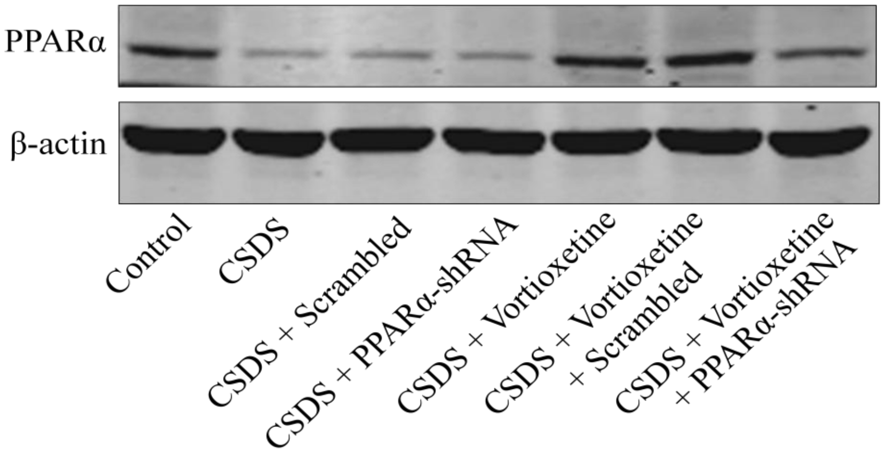
PPARα
β-actin
Control
CSDS
CSDS + Scrambled
CSDS + PPARα-shRNA
CSDS + Vortioxetine
CSDS + Vortioxetine + Scrambled
CSDS + Vortioxetine + PPARα-shRNA

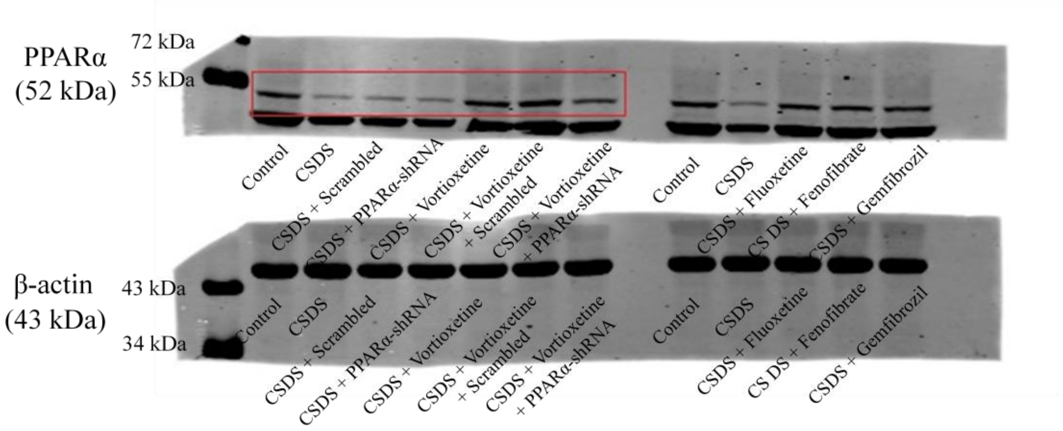
PPARα (52 kDa)
72 kDa
55 kDa
β-actin (43 kDa)
43 kDa
34 kDa
Control
CSDS
CSDS + Scrambled
CSDS + PPARα-shRNA
CSDS + Vortioxetine
CSDS + Vortioxetine + Scrambled
CSDS + Vortioxetine + PPARα-shRNA
Control
CSDS
CSDS + Fluoxetine
CS DS + Fenofibrate
CSDS + Gemfibrozil

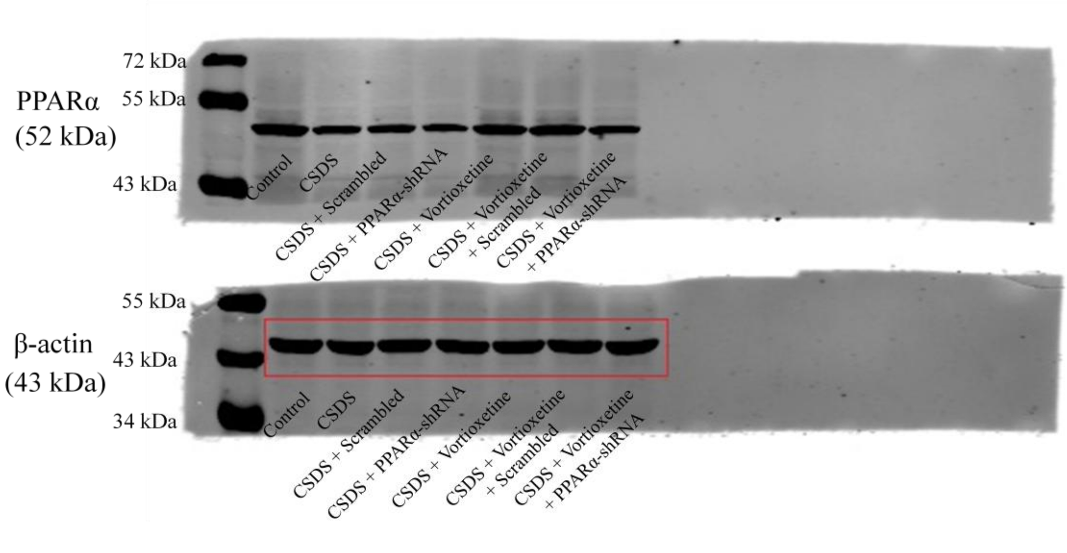
PPARα (52 kDa)
72 kDa
55 kDa
43 kDa
β-actin (43 kDa)
55 kDa
43 kDa
34 kDa
Control
CSDS
CSDS + Scrambled
CSDS + PPARα-shRNA
CSDS + Vortioxetine
CSDS + Vortioxetine + Scrambled
CSDS + Vortioxetine + PPARα-shRNA

Figure S1

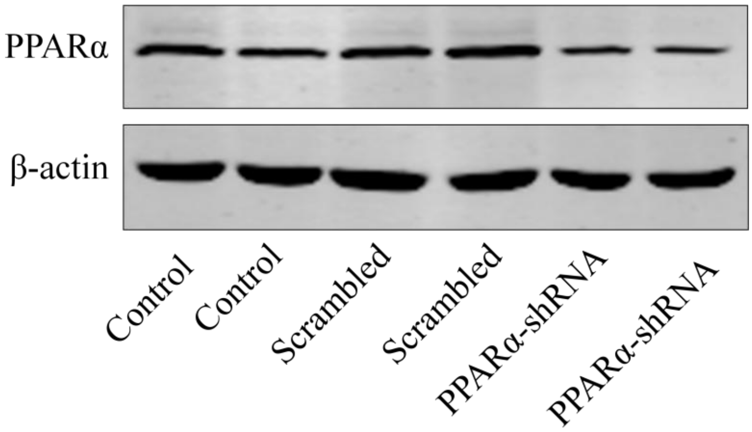
PPARα
β-actin
Control
Control
Scrambled
Scrambled
PPARα-shRNA
PPARα-shRNA

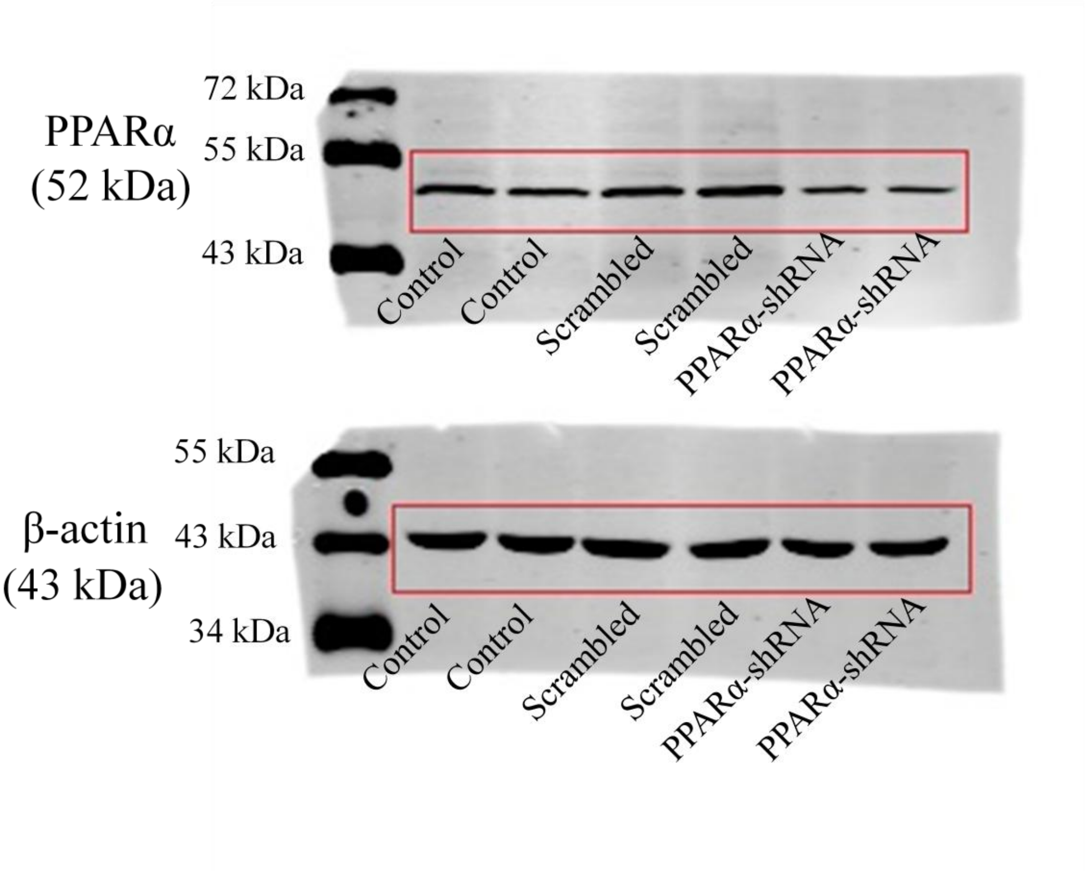
PPARα
(52 kDa)
72 kDa
55 kDa
43 kDa
Control
Control
Scrambled
Scrambled
PPARα-shRNA
PPARα-shRNA
β-actin
(43 kDa)
55 kDa
43 kDa
34 kDa
Control
Control
Scrambled
Scrambled
PPARα-shRNA
PPARα-shRNA
